# 97856 Implementation of DPYD and UGT1A1 pharmacogenetic testing to guide chemotherapy dosing

**DOI:** 10.1017/cts.2021.666

**Published:** 2021-03-30

**Authors:** Lisa A. Varughese, Kelsey S. Lau-Min, Ursina Teitelbaum, AnnaClaire Osei-Akoto, Nandi Reddy, Nevena Damjanov, Ryan Massa, Sony Tuteja

**Affiliations:** 1 University of Pennsylvania; 2 Penn Medicine at Lancaster General Health

## Abstract

ABSTRACT IMPACT: The implementation of DPYD and UGT1A1 pharmacogenetic testing, a promising tool of precision medicine, translates evidence-based research into clinical oncology practice with personalized dosing to better predict interpatient variability in chemotherapy tolerability. OBJECTIVES/GOALS: Patients with DPYD and UGT1A1 genetic variants are at risk for severe toxicity from fluoropyrimidines and irinotecan, respectively. We propose that providing clinicians with the option to order a pharmacogenetic (PGx) test with relevant dose recommendations will increase test uptake to guide pharmacotherapy decisions and improve safety outcomes. METHODS/STUDY POPULATION: We plan to conduct a non-randomized, pragmatic, open-label study in 600 adult patients with gastrointestinal (GI) cancers initiating a fluoropyrimidine- and/or irinotecan-based regimen at three cancer centers within a health system. Implementation metrics of a new, in-house laboratory developed PGx test will be measured, including feasibility of returning results within one week, fidelity of providers following dose recommendations, and penetrance via test ordering rates. Clinical aims will include assessing severe toxicity during the first six months of chemotherapy. Outcomes will be compared to a historical control of GI cancer patients enrolled in a biobank and treated with standard dose chemotherapy. RESULTS/ANTICIPATED RESULTS: We anticipate that there will be an increase in PGx test uptake given its shorter turnaround time to facilitate clinical decision-making prior to the first dose of chemotherapy. Through integration of test results in the electronic health record (EHR) and clinical decision support tools for patients with actionable genotypes, we also expect that providers will have a high level of agreement to the recommended dose adjustments. We anticipate a decreased incidence of severe (Grade >3) toxicity among prospectively genotyped patients in the first six months of chemotherapy compared to DPYD and UGT1A1 variant carriers in the historical control group. Exploratory clinical utility data on costs of hospitalizations, chemotherapy treatment, PGx test, and medical services will also be reported. DISCUSSION/SIGNIFICANCE OF FINDINGS: This study aims to address barriers identified by key stakeholders to implementing PGx testing to better tailor chemotherapy dosing to the genetic profiles to patients. This may prevent adverse event-related hospitalizations, improve quality of life for patients, and reduce health system resource utilization costs.

